# Characterization and evaluation of potential halotolerant phosphate solubilizing bacteria from *Salicornia fruticosa* rhizosphere

**DOI:** 10.3389/fpls.2023.1324056

**Published:** 2024-01-15

**Authors:** E. A. P. Teles, J. F. Xavier, F. S. Arcênio, R. L. Amaya, J. V. S. Gonçalves, L. F. M. Rouws, E. Zonta, I. S. Coelho

**Affiliations:** ^1^Laboratory of Molecular Genetics of Microorganisms, Department of Veterinary Microbiology and Immunology, Veterinary Institute, Federal Rural University of Rio de Janeiro, Seropedica, Brazil; ^2^Embrapa Agrobiologia, Seropedica, Brazil; ^3^Laboratory of Soil-Plant Relationship, Department of Soils, Institute of Agronomy, UFRRJ, Seropedica, Brazil

**Keywords:** salinity, tolerance, phosphorus, rhizobacteria, soil

## Abstract

Soil salinization is a significant abiotic factor threatening agricultural production, while the low availability of phosphorus (P) in plants is another worldwide limitation. Approximately 95–99% of the P in soil is unavailable to plants. Phosphate-solubilizing bacteria (PSB) transform insoluble phosphates into soluble forms that plants can utilize. The application of PSB can replace or partially reduce the use of P fertilizers. Therefore, selecting bacteria with high solubilization capacity from extreme environments, such as saline soils, becomes crucial. This study aimed to identify twenty-nine bacterial strains from the rhizosphere of *Salicornia fruticosa* by sequencing the 16S rDNA gene, evaluate their development in increasing concentrations of NaCl, classify them according to their salinity response, and determine their P solubilization capability. The bacteria were cultivated in nutrient agar medium with NaCl concentrations ranging from 0.5% to 30%. The phosphate solubilization capacity of the bacteria was evaluated in angar and broth National Botanical Research Institute (NBRIP) media supplemented with calcium phosphate (CaHPO_4_) and aluminum phosphate (AlPO_4_), and increased with 3% NaCl. All bacterial strains were classified as halotolerant and identified to the genera *Bacillus*, *Enterobacter*, *Halomonas*, *Kushneria*, *Oceanobacillus*, *Pantoea*, *Pseudomonas*, and *Staphylococcus*, with only one isolate was not identified. The isolates with the highest ability to solubilize phosphorus from CaHPO_4_ in the liquid medium were *Kushneria* sp. (SS102) and *Enterobacter* sp. (SS186), with 989.53 and 956.37 mg·Kg^-1^ P content and final pH of 4.1 and 3.9, respectively. For the solubilization of AlPO_4_, the most effective isolates were *Bacillus* sp. (SS89) and *Oceanobacillus* sp. (SS94), which raised soluble P by 61.10 and 45.82 mg·Kg^-1^ and final pH of 2.9 and 3.6, respectively. These bacteria demonstrated promising results in *in vitro* P solubilization and can present potential for the development of bioinput. Further analyses, involving different phosphate sources and the composition of produced organic acids, will be conducted to contribute to a comprehensive understanding of their applications in sustainable agriculture.

## 1 Introduction

Soil salinization is one of the main abiotic factors that threaten agricultural production (Gamalero et al., 2020; Sagar et al., 2022). Saline soils naturally occur in arid and semi-arid climatic regions under limited drainage conditions, associated with the presence of a high water table, and in coastal regions (Pedrotti et al., 2015; Gamalero et al., 2020; Sagar et al., 2022). This process is aggravated by climate change, which causes increases in global temperature, rainfall regimes, and sea level (Ullah et al., 2021). Anthropogenic actions also cause the accumulation of salts in the soil, and it can occur in agricultural areas owing to incorrect management of irrigation water, use of low-quality water, poor drainage, and poor soil and fertilizer management (Pedrotti et al., 2015; Gamalero et al., 2020; Sagar et al., 2022). The occurrence of salinized arable land increases annually. It is estimated that more than 3.0% of the surface soils and 6% of the subsoils are salinized owing to natural or anthropogenic processes (Food and Agriculture Organization, 2021). Salinization has accelerated in coastal agricultural lands, with salinity increasing from 1 to 33% over the last 25 years (Ullah et al., 2021).

Most plant species cannot tolerate high salinity (Etesami and Beattie, 2018; Ondrasek et al., 2022). The harmful effects caused by excess salt in plants are related to osmotic and ionic stress, which globally affect the plant and impair its water balance and nutrition, leading to a reduction in the photosynthetic rate and the generation of reactive oxygen species that cause molecular damage (Hashem et al., 2016; Bulgari et al., 2019). Thus, soil salinization severely affects crop productivity and limits agricultural use in affected areas. However, some plant species, called halophyte plants, have adapted to environments with high salinity (Etesami and Beattie, 2018). These adaptations can be morphological, physiological, or biochemical but can also be related to symbiotic interactions with plant growth-promoting bacteria (PGPB) (Etesami and Beattie, 2018; Egamberdieva et al., 2022). Plants of the genus *Salicornia* are halophytes that develop in coastal regions and have ecological importance and commercial value, as they develop in areas where most plants cannot develop and produce a large amount of biomass that can be used for the production of vegetable salt (Furtado et al., 2019). Some studies have reported PGPB isolated from the rhizosphere of *Salicornia* spp., such as *Klebsiella pneumoniae* from *Salicornia bigelovii* (Rueda-Puente et al., 2003), *Pseudomonas pseudoalcaligenes* from *Salicornia europea* (Ozawa et al., 2007) and *Brachybacterium sausashtrense* and *Pseudomonas* spp. from *Salicornia brachiata* (Jha et al., 2012).

Another limiting factor in agricultural production worldwide is the low availability of phosphorus (P) in plants. Plants absorb P in the form of monobasic (H_2_PO_4_) and dibasic (HPO_4_^2-^) ions (Gomes et al., 2010). However, approximately 95–99% of the P in soil is unavailable to plants. It is associated with the mineral fraction, mainly calcium in calcareous soils, iron and aluminum in acidic soils, and organic compounds in soils rich in organic matter (Gomes et al., 2010; Rasul et al., 2019). In acidic soils, the most common forms are aluminum phosphates variscite (AlPO_4_•2H_2_O), followed by strengite (FePO_4_•2H_2_O) (Bashan et al., 2013). In alkaline soils with high availability of Ca^+^, phosphate is associated with calcium (P-Ca), which are, in decreasing order of solubility, dihydrated dicalcium phosphate (brushite) CaHPO_4_•2H_2_O > anhydrous dicalcium phosphate (monetite) CaHPO_4_ > octacalcium phosphate Ca_8_H_2_(PO_4_)_6_•5H_2_O > tricalcium phosphate Ca_3_(PO_4_)_2_ > hydroxyapatite Ca_5_(PO_4_)_3_OH > fluorapatite Ca_5_(PO_4_)_3_F (Bashan et al., 2013). It is estimated that more than 40% of agricultural land has limited productivity due to P deficiency (Balemi and Negisho, 2012). In recent years, the use of phosphate fertilizers to maintain agricultural production has increased. For example, in Brazil, the total annual use of phosphate fertilizers has increased from an annual average of 0.04 T in 1960 to 2.2 T in 2016 (Withers et al., 2018). However, P added to the soil is quickly immobilized and becomes inaccessible to plants, resulting in the low utilization of phosphate fertilizer. In addition, the application and accumulation of phosphate fertilizers promote the eutrophication of water bodies and contamination by metals in the soil, causing damage to plants, animals, and humans (Katherine, 2010; Azzi et al., 2017). Additionally, the high salinity may induce competition between H_2_PO_4_^−^ and Cl^−^ ions (Maksimovic and Žarko, 2012). Therefore, the consequences of saline stress can lead to even greater difficulties in phosphorus uptake by plants. Moreover, plants under salt stress are most affected in crucial systems for nutrient absorption, cellular membrane stability, and transport pathways, and this also affects the absorption of phosphorus by the plant (Muhammed et al., 1987; Cruz et al., 2018; Roy and Chowdhury, 2021).

Phosphate-solubilizing microorganisms play a crucial role in the dynamics of P cycling in the soil (Alori et al., 2017, Zhu et al., 2018). These microorganisms can act as plant growth promoters by making P available to plants (Oliveira et al., 2009). The mobilization of P by microorganisms occurs through a variety of mechanisms such as a) acidification of the medium by the extrusion of H^+^ and/or organic acids, b) metal complexation, c) metal reduction, d) extrusion of phosphatases, and e) indirect dissolution of phosphate through the stimulation of acid production by plants (Bashan et al., 2013; Krishnaraj and Dahale, 2014). Various studies have sought to isolate and identify phosphate-solubilizing bacteria as bioprospecting strains with the potential to develop sustainable alternatives for P management in agriculturalOliveira et al., 2009; Zhu et al., 2011; Jiang et al., 2018; Suleman et al., 2018; Chen and Liu, 2019; Wan et al., 2020; Jiang et al., 2022). Many soil microorganisms can mobilize P; however, their transformation capacity may be associated with ecological conditions.

Environmental stressors lead to a decrease in various microbial activities, such as respiration, nitrogen mineralization, and the functioning of various enzymes (Zhu et al., 2011; Singh, 2016). An increase in salinity can profoundly impact the efficacy of phosphate-solubilizing microorganisms due to the inhibition of certain enzyme activities (Sritongon et al., 2022). For instance, the activities of enzymes such as dehydrogenases, which play a crucial role in acid synthesis, and phosphatases, involved in the mineralization of organic phosphorus, can be inhibited (Silva et al., 2007; Suleman et al., 2018). As a result, the overall performance of microorganisms in phosphate solubilization can be severely compromised. Silva et al. (2007) reported that continuous soil irrigation with reject water from a salt mine decreased the activity of several enzymes, including dehydrogenases and phosphatases. On the other hand, Sritongon et al. (2022) observed high enzymatic activity in the rhizosphere soils of *O. sativa* grown in saline fields.

The prospecting of phosphate-solubilizing bacteria (PST) as an environmentally friendly alternative to improve phosphorus uptake by plants is promising. However, these bacteria may experience a reduction in their solubilization activity due to an increase in salinity. Therefore, studies aiming to bioprospect microorganisms that are tolerant to higher salt concentrations and possess attributes for plant growth are promising. Gil et al. (2023) isolated and identified bacteria from hypersaline and hypergypsic soils that exhibit traits promoting plant growth. They were capable of increasing root size under osmotic stress in *Medicago* sp. plants. In light of these considerations, this study aimed to identify and evaluate the tolerance of bacteria isolated from the rhizosphere of *Salicornia fruticosa* to increasing amounts of salt (NaCl) and their ability to solubilize calcium (CaHPO_4_) and aluminum phosphate (AlPO_4_).

## 2 Materials and methods

### 2.1 Origin of the isolates

The bacterial strains characterized in this study were isolated from the rhizosphere of *S. fruticosa* in two different saline environments: hypersaline plains (Rio de Janeiro, RJ) (23°00′10″ S, 43°34′30″ W) and a deactivated salt mine (São Pedro da Aldeia, RJ) (22°49′59″ S, 42°05′15″ W) by Xavier (2021). The bacteria were isolated in Nutrient Agar medium supplemented with 5%, 10%, 15%, and 20% NaCl. The bacteria, identified with the ‘SS’ acronym representing ‘saline soils’ from where they were isolated, have been stored in agar stock at -20°C in the Molecular Genetics Laboratory of Microorganisms at the Federal Rural University of Rio de Janeiro.

### 2.2 DNA extraction, amplification, and sequencing of the 16S rDNA gene

DNA was extracted as described by Tito et al. (2015). The 16S rDNA gene was amplified using the polymerase chain reaction (PCR) technique, using primers 338F 5’- AGAGTTTGATCCTGGCTCAG-3’ and 1378R 5’-CGGTGTGTACAAGGCCCGGGAACG-3’. PCR assays were performed in 25 µL volumes containing the following reagents: reaction buffer (1X), 1 U of Taq DNA polymerase, 2.5 mM of MgCl_2_, 0.2 mM of dNTP, and 0.4 µM of each primer. The reaction was performed in a thermocycler (Bio-Rad, Hercules, CA, USA), with an initial step of denaturation at 94°C for 5 min; followed by 35 cycles of denaturation at 94°C for 30 s, annealing at 56°C for 40 s; extension at 72°C for 1 min and 30 s; and a final extension step at 72°C for 7 min (Weisburg et al., 1991). The PCR products were separated using electrophoresis on a 1.5% agarose gel, stained with SYBR Green I (Life Technologies, Carlsbad, CA, EUA), and visualized under ultraviolet light using an L-PIX EX photodocumentation system (Loccus Biotechnology, Cotia, SP, Brazil).

The PCR products were purified using Exo-Sap (USB Corporation, Cleveland, Ohio, USA), as recommended by the manufacturer. The purified PCR products were sequenced using BigDye™ Terminator v3.1 Cycle Sequencing Kit. The reaction was performed in a thermocycler (Bio-Rad, Hercules, CA, USA), with initial denaturation at 94°C for 1 min; followed by 35 cycles of denaturation at 94°C for 15 s, annealing at 56°C for 15 s; and extension at 72°C for 4 min. Samples were purified by precipitation using 3M sodium acetate, 125 mM EDTA, and 70% ethanol. Sequencing of the samples was performed using the 3500 Genetic Analyzer equipment (Applied Biosystems®).

The sequences were edited using BioNumerics software (v. 7.6) and compared with the sequences deposited in the NCBI database using the BLASTn algorithm (Altschul et al., 1997). Sequence alignment was performed using the ClustalW algorithm (Thompson et al., 1994) in MEGAX software (v.11.0.8). Phylogenetic relationships were determined using the neighbor joining (NJ) algorithm and p-distance model. The strength of each branch was determined using a nonparametric bootstrap test with 1000 repetitions (Felsenstein, 1985). A sequence derived from *Arthrobacter oryzae* (NR 041545.1) was used as the external group.

### 2.3 Salinity test and classification of bacterial isolates

Bacterial isolates were seeded onto nutrient agar (meat extract 1.0 g.L^-1^; yeast extract 2.0 g.L^-1^; peptone 5.0 g.L^-1^; sodium chloride 5.0 g.L^-1^; agar 15. 0 g.L^-1^) with increasing concentrations of NaCl (0.5%, 1%, 1,5%, 2%, 2,5%, 3%, 5%, 10%, 15%, 20%, 25%, and 30%). Plates containing the cultures were placed in an incubator at 30°C and analyzed over 10 days after inoculation. The growth interval was determined based on the NaCl concentration at which the isolate grew. The bacteria were inoculated into the medium in triplicates.

The isolates were classified according to their growth intervals at different NaCl concentrations: non-halophilic (0.5% to 3% NaCl), halotolerant (0.5 to > 5% NaCl), and halophilic (5% to 25%). The classification range was adapted from Oren (2013) and Daoud and Ben Ali (2020).

### 2.4 Evaluation of phosphorus-solubilizing ability in solid medium

The bacterial isolates were inoculated into test tubes containing 5 mL of DYGS medium and incubated for 24 h at 150 rpm. Subsequently, the optical density (OD) of each culture was adjusted to 0.9–1.0 by spectrophotometry at 600 nm. An aliquot of 7 µL of the cultures was inoculated onto 6 mm paper discs arranged on modified National Botanical Research Institute’s phosphate (NBRIP) medium (glucose 10 gL^-1^; MgCl_2_.6H_2_O 5 gL^-1^; MgSO_4_.7H_2_O, 0.25 gL^-1^; KCl, 0.2 gL^-1^ and (NH_4_)_2_SO_4_, 0.1 g L^-1^; plus 3% NaCl and containing 5gL^-1^ of CaHPO_4_ or AlPO_4_ as a phosphate source. The bacteria were inoculated into the medium in triplicates. The solubilization halo formed around the colonies was determined on the seventeenth day after inoculation. The solubilization index (SI) was calculated using the following formula:


$$SI=\frac{halo\;diameter\;\left(mm\right)}{colony\;diameter\;\left(mm\right)}$$

The bacterial isolates were classified according to their solubilization capacity as low (SI<2), medium (2≤SI<4), and high (SI≥4) (Berraquero et al., 1976).

### 2.5 Evaluation of phosphorus-solubilizing ability in liquid medium

The bacterial isolates were cultured and their optical densities were adjusted as previously described. The NBRIP liquid culture medium was supplemented with 3% NaCl containing CaHPO_4_ or AlPO_4_ and the pH was adjusted to 7.0. The isolates were inoculated into the medium in triplicates. Three hundred microliters of each culture was inoculated into 50 mL falcon tubes containing 35 mL of NBRIP medium and cultivated under agitation at 150 rpm for 14 days. The concentration of soluble phosphate was determined on the day of inoculation (day 0) and at the end of the incubation period (final 14 days). For this, a 10 mL aliquot of each sample was transferred to a 15 mL falcon tube and centrifuged at 6000 rpm for 10 min. The supernatant was filtered with the aid of a syringe filter with a 0.22-µm membrane. The filtrate was used to determine the soluble phosphate content with adapted methods described by Teixeira et al. (2017), and the final pH of the medium after cultivation. For quantification, the filtrates were diluted 1:150 (*v/v*) or 1:200 (*v/v*) for CaHPO_4_ and 1:50 for AlPO_4_ using deionized water. Quantification is based on the formation of a blue-colored molybdic phosphorus complex obtained after the reduction of molybdate with ascorbic acid and measured by spectrophotometry at 660 nm. A phosphate standard curve was constructed using anhydrous KH_2_PO_4_. Available P values were determined according to the equation:


$$P=\frac{L-b}{a}*d$$

In which:

P – concentration of available phosphorus, in mg kg^-1^.
L – sample absorbance reading.
a – angular coefficient of the standard curve (intercept).
b – linear coefficient of the standard curve.
d – dilution factor of the filtrate.

## 3 Results

### 3.1 Identification of bacteria and classification according to salinity

**Figure 1:**
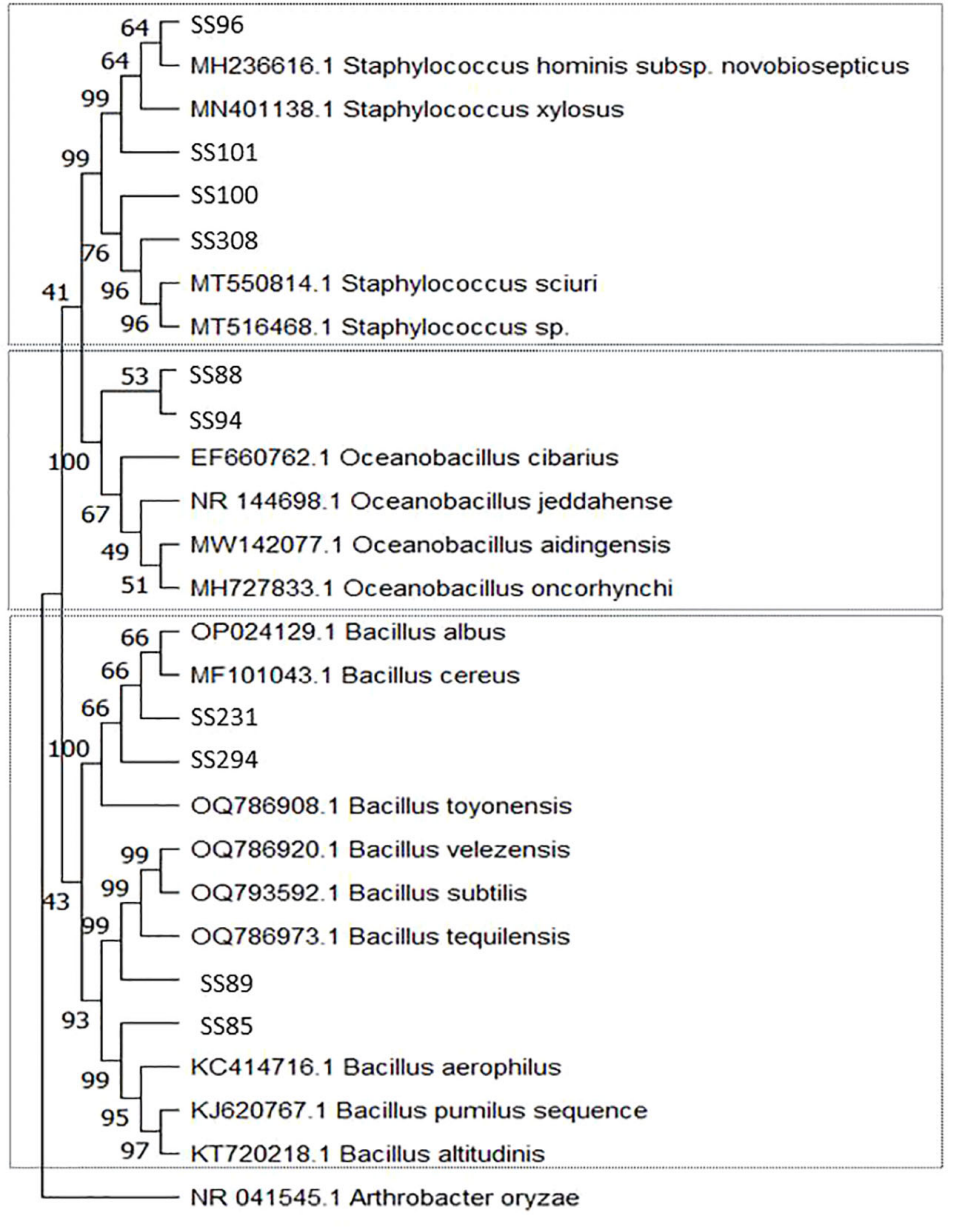
Phylogenetic tree constructed by the Neighbor-Joining method and Tajima-Nei model based on the 16S rDNA gene sequences of Proteobacteria phylum bacteria isolated from the rhizosphere of *Salicornia fruticosa*. The numbers at the nodes indicate the bootstrap values from 1,000 replicas.

**Figure 2:**
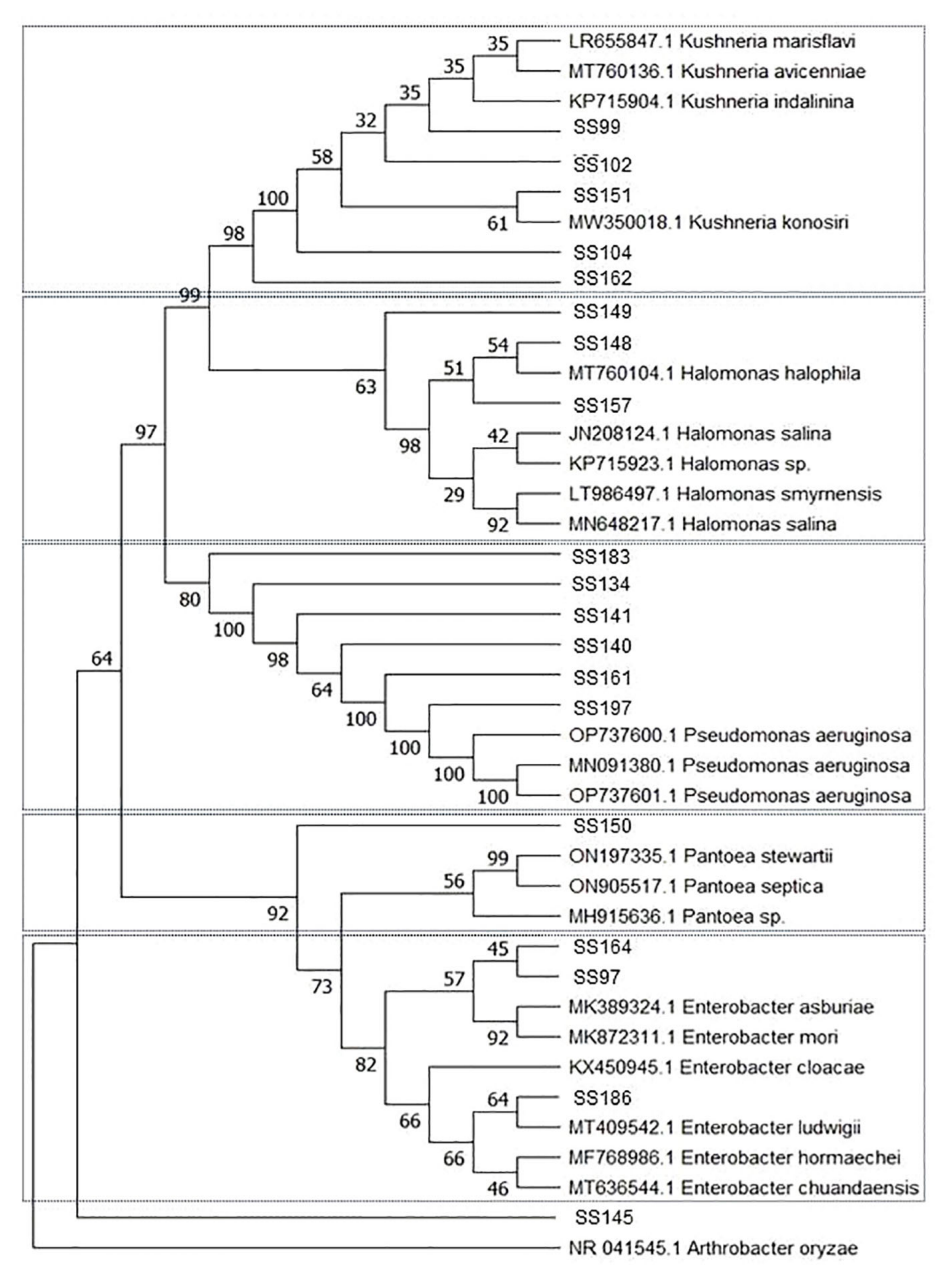
Phylogenetic tree constructed by the Neighbor-Joining method and Tajima-Nei model based on the 16S rDNA gene sequences of Firmicutes phylum bacteria isolated from the rhizosphere of *Salicornia fruticosa*. The numbers at the nodes indicate the bootstrap values from 1,000 replicas.

Among the 29 bacterial strains, 19 belonged to the phylum Proteobacteria and 10 belonged to the phylum Firmicutes. Bacteria in Proteobacteria belonged to the genera *Enterobacter*, *Halomonas*, *Kushneria*, *Pantoea*, and *Pseudomonas*. Isolate (SS145) grouped with isolates from the phylum Proteobacteria but was not associated with any clade (**Table 1**, **Figure 1**). Isolates from the phylum Firmicutes belonged to the genera *Bacillus*, *Oceanobacillus*, and *Staphylococcus* (**Table 1**, **Figure 2**).

**Table 1:** Identification of isolates, collection area, percentage of salt in the isolation medium, growth range, classification according to salinity, solubilization index (SI) in solid culture medium with CaHPO_4,_ and quantification of solubilized P and final pH value in liquid culture medium containing CaHPO_4_ and AlPO_4_.

Isolate ID	Genus	Collection area	%NaCl in the isolation medium	Growth interval	Classification according to salinity	SI	CaHPO_4_		AlPO_4_	
							P (mg kg^-1^)	pH	P (mg kg^-1^)	pH
SS85	*Bacillus* sp./CP049019.1	Deactivated salt mine	5%	0.5% - 10%	Halotolerant	Medium	474.798	4.2	2.50	2.8
SS89	*Bacillus* sp./CP049019.1	Deactivated salt mine	5%	0.5% - 10%	Halotolerant	Low	210.651	4.5	61.10	4.3
SS231	*Bacillus* sp./CP115738.1	Hypersaline plains	5%	0,5% - 5%	Halotolerant	Medium	500.476	4.5	1.84	2.9
SS294	*Bacillus* sp./KX456341.1	Hypersaline plains	5%	0,5% - 10%	Halotolerant	Medium	351.641	4.7	8.02	3.8
SS186	*Enterobacter* sp./KR189294.1	Hypersaline plains	5%	0,5% - 15%	Halotolerant	Medium	956.372	3.9	12.36	4.7
SS97	*Enterobacter* sp./MK872311.1	Deactivated salt mine	15%	0,5% - 15%	Halotolerant	Medium	327.984	4.8	5.29	5.0
SS164	*Enterobacter* sp./MT613378.1	Hypersaline plains	5%	0,5% - 5%	Halotolerant	Medium	648.122	4.2	7.01	4.7
SS148	*Halomonas* sp./KP715923.1	Hypersaline plains	15%	0,5% - 15%	Halotolerant	Low	553.733	4.1	3.27	3.5
SS149	*Halomonas* sp./KY436502.1	Hypersaline plains	15%	0,5% - 15%	Halotolerant	Medium	213.861	4.7	7.91	3.2
SS157	*Halomonas* sp./MT760104.1	Hypersaline plains	5%	0,5% - 15%	Halotolerant	Low	401.450	4.5	9.75	3.6
SS151	*Kushneria* sp./AB970650.1	Hypersaline plains	15%	0,5% - 15%	Halotolerant	Low	266.286	4.4	9.75	3.8
SS162	*Kushneria* sp./KF560351.1	Hypersaline plains	5%	0,5% - 10%	Halotolerant	Medium	587.732	4.3	5.41	3.7
SS104	*Kushneria* sp./LR655847.1	Deactivated salt mine	20%	0,5% - 20%	Halotolerant	Low	345.340	4.3	2.50	3.8
SS99	*Kushneria* sp./NR_044001.1	Deactivated salt mine	15%	0,5% - 20%	Halotolerant	Medium	274.251	5.0	13.02	4.0
SS102	*Kushneria* sp./NR_044001.1	Deactivated salt mine	20%	0,5% - 20%	Halotolerant	Medium	989.539	4.1	10.88	3.0
SS88	*Oceanobacillus* sp./MH118526.1	Deactivated salt mine	5%	0,5% - 10%	Halotolerant	Medium	310.390	4.1	4.22	3.4
SS94	*Oceanobacillus* sp./MH118526.1	Deactivated salt mine	5%	0,5% - 20%	Halotolerant	Low	218.141	5.1	45.83	3.6
SS150	*Pantoea* sp./MH915636.1	Hypersaline plains	15%	0,5% - 20%	Halotolerant	Medium	436.044	4.6	23.89	3.5
SS141	*Pseudomonas* sp./KY072850.1	Deactivated salt mine	5%	0,5% - 10%	Halotolerant	Low	410.010	4.7	6.30	3.2
SS183	*Pseudomonas* sp./MN625859.1	Hypersaline plains	5%	0,5% - 15%	Halotolerant	Low	376.486	4.2	21.28	3.0
SS134	*Pseudomonas* sp./OP737584.1	Deactivated salt mine	15%	0,5% - 15%	Halotolerant	Medium	485.616	3.9	9.21	4.8
SS140	*Pseudomonas* sp./OP737584.1	Deactivated salt mine	5%	0,5% - 15%	Halotolerant	Low	365.430	4.4	8.98	4.6
SS161	*Pseudomonas* sp./OP737584.1	Hypersaline plains	5%	0,5% - 5%	Halotolerant	Medium	292.796	4.7	3.39	4.6
SS197	*Pseudomonas* sp./OP737601.1	Hypersaline plains	5%	0,5% - 5%	Halotolerant	Medium	687.589	5.0	4.22	3.4
SS96	*Staphylococcus* sp./LC511705.1	Deactivated salt mine	15%	0,5% - 15%	Halotolerant	Medium	344.270	4.7	7.55	3.6
SS101	*Staphylococcus* sp./MT353655.1	Deactivated salt mine	15%	0,5% - 15%	Halotolerant	Low	466.358	4.8	11.12	4.8
SS100	*Staphylococcus* sp./MT550814.1	Deactivated salt mine	15%	0,5% - 15%	Halotolerant	Low	532.573	4.7	14.68	4.3
SS308	*Straphylococcus* sp./MT550814.1	Hypersaline plains	5%	0,5% - 15%	Halotolerant	Medium	240.490	4.6	12.60	3.7
SS145	Uncultured bacterium/HQ143327.1	Hypersaline plains	15%	0,5% - 10%	Halotolerant	Low	783.286	5.6	1.84	4.3

All isolates grown in both media containing 0.5% and above 5% NaCl, than were classified as halotolerant (**Table 1**) (Oren, 2008; Daoud and Ben Ali, 2020). Two isolates of *Pseudomonas* spp., one of *Enterobacter* sp., and one of *Bacillus* sp. developed at a maximum concentration of 5% NaCl. Three isolates of *Bacillus* spp., one of *Kushneria* sp., one of *Pseudomonas* sp., one of *Oceanobacillus* sp., and isolate (SS145) grew in 10% NaCl. Four isolates of *Staphylococcus* spp., three of *Halomonas* spp., three of *Pseudomonas* spp., two of *Enterobacter* spp., one of *Bacillus* sp., and one of *Kushneria* sp. accounted for growth up to 15% NaCl. Finally, three isolates of *Kushneria* spp., one of *Pantoea* sp., and one of *Oceanobacillus* sp. were grown in media containing up to 20% NaCl.

### 3.2 Phosphate solubilization in solid medium

**Figure 3:**
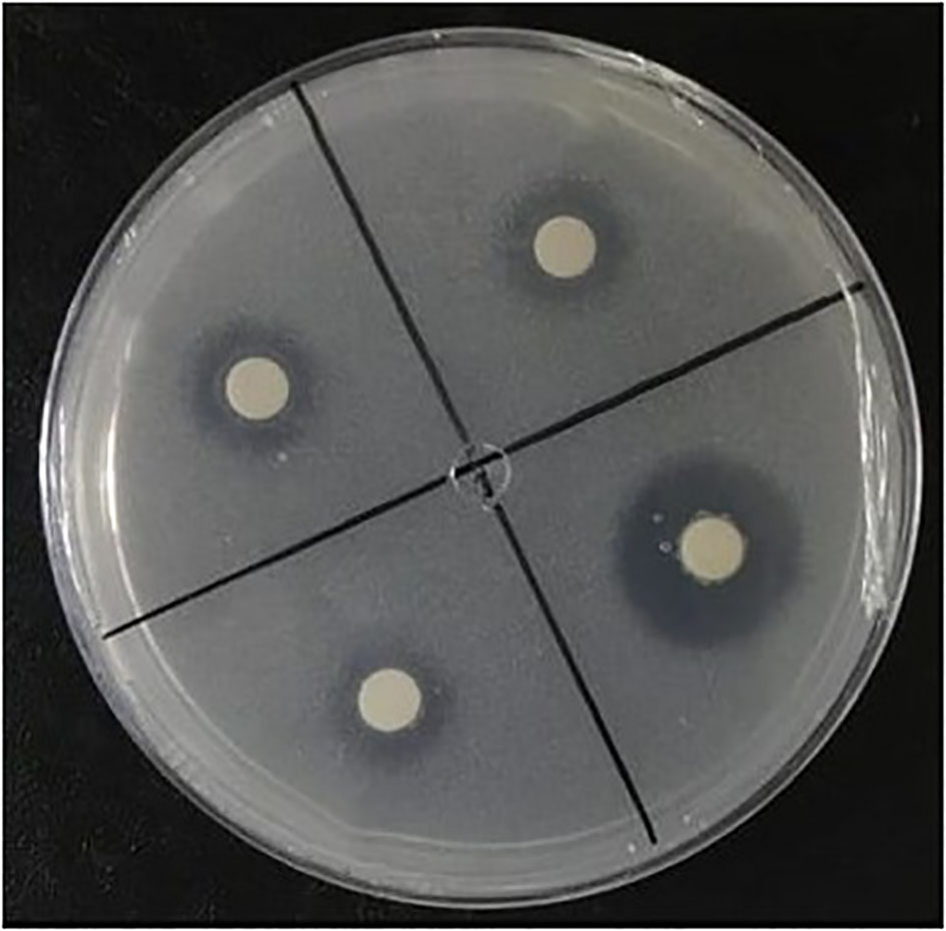
NBRIP medium containing CaHPO_4_ inoculated with bacteria, displaying solubilization halos.

All isolates grew in a solid culture medium containing dibasic calcium phosphate (CaHPO_4_) as a phosphate source (**Figure 3**). Ten isolates (31.0%) showed a low solubilization and 18 (58.6%) showed a medium SI (**Table 1**). Despite colony development, halo formation was not observed in the isolates of *Pseudomonas* sp. (SS140) and *Halomonas* sp. (SS157). The isolates also grew in a culture medium containing aluminum phosphate (AlPO_4_) as a P source, but no halo formation was observed.

### 3.3 Phosphate solubilization in liquid medium

**Figure 4:**
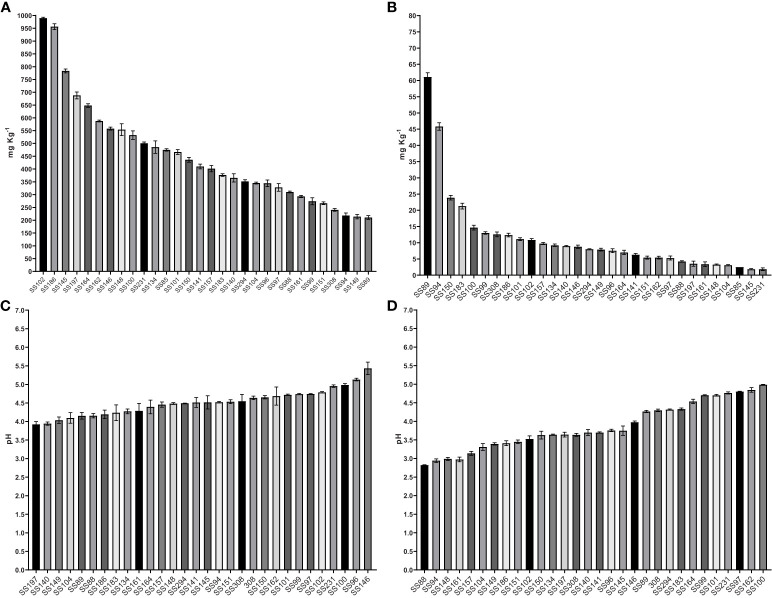
The P solubilizing levels, in mg Kg^-1^, of bacteria in liquid NBRIP medium containing CaHPO_4_ **(A)**, AlPO_4_ **(B)**, and final pH on medium containing CaHPO_4_ **(C)** and AlPO_4_ **(D)**. The results are the mean value of three replicates, error bars represent standard error.

The solubilization of the two sources of phosphorus, CaHPO_4_ and AlPO_4_, by the bacterial isolates was accompanied by a reduction in pH. The content of P solubilized from CaHPO_4_ varied from 210.65 to 989.54 mg·Kg^-1^ and the pH of the culture media at the end of cultivation varied from 5.4 to 3.9 (**Table 1**, **Figure 4**). The *Kushneria* sp. (SS102) demonstrated the highest CaHPO_4_ solubilization, reaching 989.54 mg•Kg^-1^, resulting in a final pH of 4.1. An increase in available P was also observed in media containing AlPO_4_, varying from 1.84 to 61.10 mg·Kg^-1^. The decrease in pH in media containing AlPO_4_ was higher than that in media containing CaHPO_4_, varying from 5.0 to 2.8 (**Table 1**, **Figure 4**). The *Bacillus* sp. (SS89) and *Oceanobacillus* sp. (SS94) isolates showed the highest soluble P contents for AlPO_4_, 61.10 and 45.83 mg·Kg^-1^, with a final pH of 2.9 and 3.6.

## 4 Discussion

The inoculation of cultivated plants with PGPB is considered a promising practice for promoting the development of agriculture under adverse conditions, such as saline soils (Gamalero et al., 2020). The main objective of this study was to evaluate the efficiency of halotolerant bacterial isolates from the rhizosphere of the halophyte *S. fruticosa* in the inorganic phosphates solubilization in solid and liquid culture media. The isolation and identification of bacteria associated with halophyte plants with growth-promoting attributes can contribute to the identification of strains with the potential for the development of bioinputs (Oliveira et al., 2009).

The isolates analyzed in this study came from media containing 5–20% NaCl and were all classified as halotolerant, exhibiting growth both at a concentration of 0.5% and above 5% NaCl (Oren, 2013; Daoud and Ben Ali, 2020). The salt content in saline soils is quite variable because of the influence of environmental factors, such as rain and tidal variations, which provide microhabitats with different salt concentrations (Quesada et al., 1982; Ventosa et al., 2008). Heterogeneity in the habitat can favor the selection of halotolerant microorganisms, as they are capable of adapting to fluctuating saline conditions in these environments, which gives them an advantage over specialized microorganisms, such as halophiles. The variation in salt content may explain why halotolerant bacteria were predominant among the evaluated bacteria. Halotolerant bacteria may offer increased benefits in agriculture owing to their superior adaptability to variations in salinity compared with halophilic bacteria.

The selection of phosphate-solubilizing bacteria in solid culture media has served as a universal indicator of phosphate solubilization for over half a century because it is a simple and inexpensive technique (Bashan et al., 2013). This method is based on the formation of a translucent halo around colonies. Halo formation occurs due to the dissolution of insoluble phosphate (Katznelson et al., 1962). The exudation of organic acids is one of the main mechanisms by which bacteria mobilize mineral phosphates (Alori et al., 2017). These acids can form metallic complexes or chelates with Ca, Al, and Fe^+3^ ions associated with phosphates without leading to the formation of translucent halos in the culture media (Merbach et al., 2009; Bashan et al., 2013). Therefore, plate tests must be complemented using quantitative tests in a liquid culture medium. Among the isolates studied, 27 showed solubilization activity of CaHPO_4_ from the formation of translucent halos (**Table 1**, **Figure 3**). No solubilization halos were observed in media containing AlPO_4_ despite colony development.

All isolates showed variations in the amount of available P and a reduction in pH in the liquid medium tests with CaHPO_4_ and AlPO_4_ (**Table 1**, **Figure 3**). Thus, solid culture media may be more suitable for the isolation of phosphate-solubilizing bacteria, as the ability to develop colonies in media containing insoluble P sources may indicate solubilization capacity even in the absence of halo formation. The levels of P mobilized from CaHPO_4_ were higher than those mobilized from AlPO_4_. The solubility of P-Ca was strongly influenced by pH, increasing rapidly with a decrease in pH from 6.5. However, the solubility of P–Al increases because of acids at pH levels below 3, which is rarely observed in soils (Fankem et al., 2008). Wan et al. (2020) observed similar results in tests carried out with 18 phosphate-solubilizing bacteria isolated from soil at the Laiyang Experimental Station, Shandong, China, which were able to solubilize from 47.08 to 250.77 mg L^-1^ of P from Ca_3_(PO_4_)_2_ and from 14.99 to 81.99 mg L^-1^ from AlPO_4_. Fankem et al. (2008) tested strains of *P. fluorescens* to solubilize different inorganic phosphate sources: Ca_3_(PO_4_)_2_, AlPO_4,_ and FePO_4_. Solubilization of Ca-P was possible by simply acidifying the medium. In addition, the authors analyzed the action of different organic acids produced by bacteria on Ca_3_(PO_4_)_2_ at pH 7 and 4 and found that acidity contributed to the solubilization of Ca-P by carboxylic acids. In contrast, the solubilization of AlPO_4_ and FePO_4_ seems to be related to the stability constants of the bond between the Fe or Al complex and different organic acids exuded by the bacteria (Fankem et al., 2008). Merbach et al. (2009) observed the production of various organic acids by bacteria capable of solubilizing different sources of mineral phosphate. These authors pointed out that bacterial strains that were more efficient in mobilizing P from calcium phosphate produced large amounts of citrate and tartarate, and to a lesser extent, malate. Furthermore, strains that solubilize iron and aluminum phosphates have tartarate and malate as their most important carboxylates, respectively (Merbach et al., 2009).

In our analyses, *Kushneria* sp. (SS102), *Enterobacter* sp. (SS186), and isolate (SS145) solubilized the highest amount of P from CaHPO_4_, with 989.53, 956.37, and 783.82 mg·Kg^-1^, respectively. The solubilization of phosphate from AlPO_4_ was more pronounced for the isolates *Bacillus* sp. (SS89) and *Oceanobacillus* sp. (SS94), which increased the soluble P to 61.10 and 45.82 mg·Kg^-1^, respectively. These two isolates showed relatively low calcium phosphate solubilization capacity compared to the others, at 210.65 and 218.14 mg·Kg^-1^ of P. Similar results were observed by Wan et al. (2020) for a strain of *Bacillus* sp., which solubilized about 60 mg·Kg^-1^ of P from calcium phosphate but showed considerably high capacity for iron and aluminum phosphate, at 30 mg·Kg^-1^ and 50 mg·Kg^-1^, respectively. The isolates *Pantoea* sp. (SS150) and *Pseudomonas* sp. (SS183) also showed superior aluminum phosphate solubilization activity to the others, raising the soluble P in the medium to 23.99 and 21.7 mg·Kg^-1^, respectively. They showed intermediate values for calcium phosphate, at 436.04 and 376.49 mg·Kg^-1^ of soluble P. In acidic soils, there is an increases the relative distributions of cations as H^+^ and Al^3+^, which may lead to negative effects such as the depletion of nutrients and the high solubility of Al, Fe, and Mn, causing toxicity in plants (Tian and Niu, 2015). Furthermore, soil acidification reduces P availability for plant due to fixation with acidic cations such as Al and Fe (Gomes et al., 2010; Xiao et al., 2013; Qaswar et al., 2020). Therefore, the exploration of PSB capable of solubilizing aluminum phosphate can be particularly promising for Brazilian soils.

## 5 Conclusion

Bacteria isolated from the rhizosphere of *S. fruticosa* were classified as halotolerant. Some isolates demonstrated promising results in *in vitro* P solubilization and hold potential for the development of bioinputs or for the bioprocessing of rock phosphates. Analyses involving higher salt concentrations, different phosphate sources and the composition of produced organic acids will be conducted to contribute to a comprehensive understanding of their applications in sustainable agriculture.

## Data availability statement

The original contributions presented in the study are included in the article/supplementary material, further inquiries can be directed to the corresponding author.

## Author contributions

TE: Conceptualization, Data curation, Formal analysis, Investigation, Methodology, Project administration, Supervision, Visualization, Writing – original draft, Writing – review & editing. XJ: Conceptualization, Data curation, Methodology, Writing – review & editing. AF: Investigation, Methodology, Writing – review & editing. GJ: Investigation, Methodology, Writing – review & editing. AR: Investigation, Methodology, Writing – review & editing. RL: Conceptualization, Project administration, Resources, Writing – review & editing. ZE: Conceptualization, Data curation, Formal analysis, Funding acquisition, Methodology, Resources, Supervision, Writing – review & editing. CI: Conceptualization, Data curation, Formal analysis, Funding acquisition, Project administration, Resources, Supervision, Validation, Visualization, Writing – review & editing.

## References

[B1] Alori E. T. Glick B. R. Babalola O. O. (2017). Microbial phosphorus solubilization and its potential for use in sustainable agriculture. Front. Microbiol. 8. doi: 10.3389/fmicb.2017.00971 PMC5454063 28626450

[B2] Altschul S. F. Madden T. L. Schäffer A. A. Zhang J. Zhang Z. Miller W.. (1997). Gapped BLAST and PSI-BLAST: a new generation of protein database search programs. Nucleic Acids Res. 25, 3389–3402. doi: 10.1093/nar/25.17.3389 9254694 PMC146917

[B3] Azzi V. Kanso A. Kazpard V. Kobeissi A. Lartiges B. El Samrani A. (2017). Lactuca sativa growth in compacted and non-compacted semi-arid alkaline soil under phosphate fertilizer treatment and cadmium contamination. Soil Tillage Res. 165, 1–10. doi: 10.1016/j.still.2016.07.014

[B4] Balemi T. Negisho K. (2012). Management of soil phosphorus and plant adaptation mechanisms to phosphorus stress for sustainable crop production: a review. J. Soil Sci. Plant Nutr. 12, 547–562. doi: 10.4067/S0718-95162012005000015

[B5] Bashan Y. Kamnev A. A. de-Bashan L. E. (2013). Tricalcium phosphate is inappropriate as a universal selection factor for isolating and testing phosphate-solubilizing bacteria that enhance plant growth: a proposal for an alternative procedure. Biol. Fertil. Soils 49, 465–479. doi: 10.1007/s00374-012-0737-7

[B6] Berraquero F. R. Baya A. Cormenzana A. R. (1976). Establecimiento de índices para el estudio de la solubilización de fosfatos por bacterias del suelo. Ars. Pharm. 17, 399–406. doi: 10.30827/ars

[B7] Bulgari R. Franzoni G. Ferrante A. (2019). Biostimulants application in horticultural crops under abiotic stress conditions. Agronomy 9, 306. doi: 10.3390/agronomy9060306

[B8] Chen Q. Liu S. (2019). Identification and characterization of the phosphate-solubilizing bacterium *pantoea* sp. S32 in reclamation soil in Shanxi, China. Front. Microbiol. 10. doi: 10.3389/fmicb.2019.02171 PMC6761231 31608027

[B9] Cruz J. L. Coelho E. F. Coelho Filho M. A. Santos A.A.d. (2018). Salinity reduces nutrients absorption and efficiency of their utilization in cassava plants. Cienc. Rural 48, 11. doi: 10.1590/0103-8478cr20180351

[B10] Daoud L. Ben Ali M. (2020). “Halophilic microorganisms: Interesting group of extremophiles with important applications in biotechnology and environment,” in Physiological and biotechnological aspects of extremophiles Elsevier Cambridge, UK: Academic Press), 51–64. doi: 10.1016/B978-0-12-818322-9.00005-8

[B11] Egamberdieva D. Alimov J. Shurigin V. Alaylar B. Wirth S. Bellingrath-Kimura S. D. (2022). Diversity and plant growth-promoting ability of endophytic, halotolerant bacteria associated with *tetragonia tetragonioides* (Pall.) kuntze. Plants 11, 49. doi: 10.3390/plants11010049 PMC8747539 35009054

[B12] Etesami H. Beattie G. A. (2018). Mining halophytes for plant growth-promoting halotolerant bacteria to enhance the salinity tolerance of non-halophytic crops. Front. Microbiol. 9. doi: 10.3389/fmicb.2018.00148 PMC5809494 29472908

[B13] Fankem H. Ngo L. Deubel A. Quinn J. Merbach W. Etoa F.-X.. (2008). Solubilization of inorganic phosphates and plant growth promotion by strains of *Pseudomonas fluorescens* isolated from acidic soils of Cameroon. Afr. J. Microbiol. Res. 2, 171–178. doi: 10.5897/AJMR.9000660

[B14] Felsenstein J. (1985). Confidence limits on phylogenies: an approach using the bootstrap. Evolution 39, 783–791. doi: 10.1111/j.1558-5646.1985.tb00420.x 28561359

[B15] Food and Agriculture Organization (2021) Global Map of Salt-affected Soils. Available at: https://www.fao.org/soils-portal/data-hub/soil-maps-and-databases/global-map-of-salt-affected-soils/en/#:~:text=With%20the%20current%20information%20from%20118%20countries%20covering,saline%2C%2010%25%20are%20sodic%20and%205%25%20are%20saline-sodic (Accessed 10, 2023).

[B16] Furtado B. U. Gołębiewski M. Skorupa M. Hulisz P. Hrynkiewicz K. (2019). Bacterial and fungal endophytic microbiomes of *salicornia europaea* . Appl. Environ. Microbiol. 85, 13. doi: 10.1128/AEM.00305-19 PMC6581177 31003988

[B17] Gamalero E. Bona E. Todeschini V. Lingua G. (2020). Saline and arid soils: impact on bacteria, plants, and their interaction. Biol. (Basel) 9, 116. doi: 10.3390/biology9060116 PMC7344409 32498442

[B18] Gil T. Teixeira R. Sousa A. d’Oliveira Palmeiro M. A. Cruz Coimbra de Matos A. Niza Costa M.. (2023). Isolation and characterization of culturable osmotolerant microbiota in hypersaline and hypergypsic soils as new treatment for osmotic stress in plants. Soil Syst. 7, 86. doi: 10.3390/soilsystems7040086

[B19] Gomes E. A. Souza F.A.d.e Sousa S.M.d.e Vasconcelos M.J.V.d.e Marriel I. E. Silva U.C.d.a (2010). Prospecção de Comunidades Microbianas do Solo Ativasno Aproveitamento Agrícolade Fontes de Fósforo de Baixa Solubilidade Vol. 107 (Embrapa Milho e Sorgo. Documentos), 1-34. Available at: https://www.embrapa.br/busca-de-publicacoes/-/publicacao/883157/prospeccao-de-comunidades-microbianas-do-solo-ativas-no-aproveitamento-agricola-de-fontes-de-fosforo-de-baixa-solubilidade.

[B20] Hashem A. Abd Allah E. F. Alqarawi A. A. Al-Huqail A. A. Shah M. A. (2016). Induction of Osmoregulation and Modulation of Salt Stress in *Acacia gerrardii* Benth. by Arbuscular Mycorrhizal Fungi and *Bacillus subtilis* (BERA 71). BioMed. Res. Int. 2016, 6294098. doi: 10.1155/2016/6294098 27597969 PMC5002495

[B21] Jha B. Gontia I. Hartmann A. (2012). The roots of the halophyte Salicornia brachiata are a source of new halotolerant diazotrophic bacteria with plant growth-promoting potential. Plant Soil 356, 265–277. doi: 10.1007/s11104-011-0877-9

[B22] Jiang H. Qi P. Wang T. Wang M. Chen M. Chen N.. (2018). Isolation and characterization of halotolerant phosphate-solubilizing microorganisms from saline soils. 3 Biotech. 8, 461. doi: 10.1007/s13205-018-1485-7 PMC6204131 30370202

[B23] Jiang Y. Zhao X. Zhou Y. Ding C. (2022). Effect of the phosphate solubilization and mineralization synergistic mechanism of Ochrobactrum sp. on the remediation of lead. Environ. Sci. pollut. Res. Int. 29, 58037–58052. doi: 10.1007/s11356-022-19960-y 35362889

[B24] Katherine K. (2010). Environmental impacts of agricultural technologies Vol. 65 (Evans School Policy Analysis & Research (EPAR). EPAR Brief), 1–18. Available at: https://www.researchgate.net/publication/254452871_Environmental_Impacts_of_Agricultural_Technologies_EPAR_Brief_No_65

[B25] Katznelson H. Peterson E. A. Rouatt J. W. (1962). Phosphate-dissolving microorganisms on seed and in the root zone of plants. Can. J. Bot. 40, 1181–1186. doi: 10.1139/b62-108

[B26] Krishnaraj P. U. Dahale S. (2014). Mineral phosphate solubilization: concepts and prospects in sustainable agriculture. PINSA 80, 389. doi: 10.16943/ptinsa/2014/v80i2/55116

[B27] Maksimovic I. Žarko I. (2012). Effects of salinity on vegetable growth and nutrients uptake. Irrigation systems and practices in challenging environments. Ed. Lee T. S. (London, UK: InTech), 169-190. doi: 10.5772/29976

[B28] Merbach W. Fankem H. Deubel A. (2009). “Influence of rhizosphere bacteria of African oil palm (Elaeis guineensis) on calcium, iron, and aluminum phosphate in *vitro* mobilization,” in International symposium “Root Research and Applications (Vienna, AT), 2–4. Available at: http://asrr.boku.ac.at/fileadmin/files/RRcd/session03/poster/042.

[B29] Muhammed S. Akbar M. Neue H. U. (1987). Effect of Na/Ca and Na/K ratios in saline culture solution on the growth and mineral nutrition of rice (Oryza sativa L.). Plant Soil 104 (1), 57–62. doi: 10.1007/BF02370625

[B30] Oliveira C. A. Alves V. M. C. Marriel I. E. Gomes E. A. Scotti M. R. Carneiro N. P.. (2009). Phosphate solubilizing microorganisms isolated from rhizosphere of maize cultivated in an oxisol of the Brazilian Cerrado Biome. Soil Biol. Biochem. 41, 1782–1787. doi: 10.1016/j.soilbio.2008.01.012

[B31] Ondrasek G. Rathod S. Manohara K. K. Gireesh C. Anantha M. S. Sakhare A. S.. (2022). Salt stress in plants and mitigation approaches. Plants 11. doi: 10.3390/plants11060717 PMC8950276 35336599

[B32] Oren A. (2008). Microbial life at high salt concentrations: phylogenetic and metabolic diversity. Saline Syst. 4, 2. doi: 10.1186/1746-1448-4-2 18412960 PMC2329653

[B33] Oren A. (2013). Life at high salt concentrations, intracellular KCl concentrations, and acidic proteomes. Front. Microbiol. 4. doi: 10.3389/fmicb.2013.00315 PMC3817357 24204364

[B34] Ozawa T. Wu J. Fujii S. (2007). Effect of inoculation with a strain of *Pseudomonas pseudoalcaligenes* isolated from the endorhizosphere of *Salicornia europea* on salt tolerance of the glasswort. Soil Sci. Plant Nutr. 53, 12–16. doi: 10.1111/j.1747-0765.2007.00098.x

[B35] Pedrotti A. Chagas R. M. Ramos V. C. Prata A. P. M. Lucas A. A. T. Santos P. B. S. (2015). Causas e conseqüências do processo de salinização dos solos. Rev. Eletrônica em Gestão Educação e Tecnologia Ambiental 19, 1308–1324. doi: 105902/2236117016544

[B36] Qaswar M. Dongchu L. Jing H. Tianfu H. Ahmed W. Abbas M.. (2020). Interaction of liming and long-term fertilization increased crop yield and phosphorus use efficiency (PUE) through mediating exchangeable cations in acidic soil under wheat-maize cropping system. Sci. Rep. 10, 19828. doi: 10.1038/s41598-020-76892-8 33188239 PMC7666156

[B37] Quesada E. Ventosa A. Rodriguez-Valera F. Ramos-Cormenzana A. (1982). Types and properties of some bacteria isolated from hypersaline soils. J. Appl. Bacteriology 53, 155–161. doi: 10.1111/j.1365-2672.1982.tb04671.x

[B38] Rasul M. Yasmin S. Suleman M. Zaheer A. Reitz T. Tarkka M. T.. (2019). Glucose dehydrogenase gene containing phosphobacteria for biofortification of Phosphorus with growth promotion of rice. Microbiol. Res. 223–225, 1–12. doi: 10.1016/j.micres.2019.03.004 31178042

[B39] Roy S. Chowdhury N. (2021). “Salt stress in plants and amelioration strategies: A critical review” in Abiotic stress in plants. Eds. Fahad S. Saud S. Chen Y. Wu C. Wang D. (London, UK: InTech), 1-32. doi: 10.5772/intechopen.93552

[B40] Rueda-Puente E. Castellanos T. Troyo-Dieguez E. Diaz de Leon-Alvarez J. L. Murillo-Amador B. (2003). Effects of a Nitrogen-Fixing Indigenous Bacterium (*Klebsiella pneumoniae*) on the Growth and Development of the Halophyte *Salicornia bigelovii* as a New Crop for Saline Environments. J. Agro. Crop Sci. 189, 323–332. doi: 10.1046/j.1439-037X.2003.00051.x

[B41] Sagar A. Rai S. Ilyas N. Sayyed R. Z. Al-Turki A. I. El Enshasy H. A.. (2022). Halotolerant rhizobacteria for salinity-stress mitigation: diversity, mechanisms and molecular approaches. Sustainability 14, 490. doi: 10.3390/su14010490

[B42] Silva C. D. S. Vieira R. Porto E. (2007). “Efeito da salinidade sobre a atividade enzimática em solos de região semi-árida do Brasil” in CONGRESSO VIRTUAL IBEROAMERICANO SOBRE GESTION DE CALIDAD EN LABORATORIOS, 4., 2007, Madrid (Madrid: IBEROLAB), 2007. Comunicaciones...

[B43] Singh K. (2016). Microbial and enzyme activities of saline and sodic soils. Land Degrad. Dev. 27, 706–718. doi: 10.1002/ldr.2385

[B44] Sritongon N. Sarin P. Theerakulpisut P. Riddech N. (2022). The effect of salinity on soil chemical characteristics, enzyme activity and bacterial community composition in rice rhizospheres in Northeastern Thailand. Sci. Rep. 12, 20360. doi: 10.1038/s41598-022-24902-2 36437295 PMC9701763

[B45] Suleman M. Yasmin S. Rasul M. Yahya M. Atta B. M. Mirza M. S. (2018). Phosphate solubilizing bacteria with glucose dehydrogenase gene for phosphorus uptake and beneficial effects on wheat. PloS One 13, e0204408. doi: 10.1371/journal.pone.0204408 30240432 PMC6150522

[B46] Teixeira P. C. Donagemma G. K. Fontana A. Teixeira W. G. (2017). Manual de métodos de análise de solo. Brasília DF: Embrapa. 3, 204–208.

[B47] Thompson J. D. Higgins D. G. Gibson T. J. (1994). CLUSTAL W: improving the sensitivity of progressive multiple sequence alignment through sequence weighting, position-specific gap penalties and weight matrix choice. Nucleic Acids Res. 22, 4673–4680. doi: 10.1093/nar/22.22.4673 7984417 PMC308517

[B48] Tian D. Niu S. (2015). A global analysis of soil acidification caused by nitrogen addition. Environ. Res. Lett. 10, 24019. doi: 10.1088/1748-9326/10/2/024019

[B49] Tito T. M. Rodrigues N. D. M. B. Coelho S. M. O. Souza M. M. S. Zonta E. Coelho I. S. (2015). Choice of DNA extraction protocols from Gram negative and positive bacteria and directly from the soil. Afr. J. Microbiol. Res. 9, 863–871. doi: 10.5897/AJMR2014.7259

[B50] Ullah A. Bano A. Khan N. (2021). Climate change and salinity effects on crops and chemical communication between plants and plant growth-promoting microorganisms under stress. Front. Sustain. Food Syst. 5. doi: 10.3389/fsufs.2021.618092

[B51] Ventosa A. Mellado E. Sanchez-Porro C. Marquez M. C. (2008). “Halophilic and Halotolerant Micro-Organisms from Soils,” in Microbiology of extreme soils Soil Biology. Eds. Dion P. Nautiyal C. S. (Berlin, Heidelberg: Springer Berlin Heidelberg), 87–115.

[B52] Wan W. Qin Y. Wu H. Zuo W. He H. Tan J.. (2020). Isolation and characterization of phosphorus solubilizing bacteria with multiple phosphorus sources utilizing capability and their potential for lead immobilization in soil. Front. Microbiol. 11. doi: 10.3389/fmicb.2020.00752 PMC7190802 32390988

[B53] Weisburg W. G. Barns S. M. Pelletier D. A. Lane D. J. (1991). 16S ribosomal DNA amplification for phylogenetic study. J. bacteriology 173, 697–703. doi: 10.1128/jb.173.2.697-703.1991 PMC207061 1987160

[B54] Withers P. J. A. Rodrigues M. Soltangheisi A. de Carvalho T. S. Guilherme L. R. G. Benites V.. (2018). Transitions to sustainable management of phosphorus in Brazilian agriculture. Sci. Rep. 8, 2537. doi: 10.1038/s41598-018-20887-z 29416090 PMC5803245

[B55] Xavier J. F. (2021). Isolamento e Caracterização de Bactérias Associadas À Rizosfera de Plantas Halófitas. [Dissertation] ([Seropédica (RJ)]: Rural Federal University of Rio de Janeiro).

[B56] Xiao C. Chi R. Hu L. (2013). Solubilization of aluminum phosphate by specific Penicillium spp. J. Cent. South Univ. 20, 2109–2114. doi: 10.1007/s11771-013-1714-5

[B57] Zhu F. Qu L. Hong X. Sun X. (2011). Isolation and characterization of a phosphate-solubilizing halophilic bacterium kushneria sp. YCWA18 from daqiao saltern on the coast of yellow sea of China. Evid. Based Complement. Alternat. Med. 2011, 615032. doi: 10.1155/2011/615032 21716683 PMC3118493

[B58] Zhu J. Li M. Whelan M. (2018). Phosphorus activators contribute to legacy phosphorus availability in agricultural soils: A review. Sci. Total Environ. 612, 522–537. doi: 10.1016/j.scitotenv.2017.08.095 28865270

